# The use of Ligasure Vessel Sealing System in Ivor Lewis esophagectomy

**DOI:** 10.1186/1749-8090-7-10

**Published:** 2012-01-24

**Authors:** Fuat Sayir, Ufuk Çobanoğlu, Abidin Şehitoğulları

**Affiliations:** 1 Department of Thoracic Surgery, Faculty of Medicine, Yuzuncu Yil University, Van, Turkey; 2 Department of Thoracic Surgery, Faculty of Medicine, Training and Education Hospital, Van, Turkey

## Abstract

**Background:**

This study investigated the results of the LigaSure Vessel Sealing System (LVSS), which has been routinely used in esophageal resections in our clinic since 2006.

**Methods:**

For this purpose, 60 patients who underwent Ivor Lewis esophagectomy were included in the study. The results were compared with the patients who underwent stomach mobilising procedure and esophagectomy with conventional methods (conventional group) before 2006 and the patients who underwent LVSS (group of LigaSure) in surgical cases after 2006. The cases were compared particularly in terms of intraoperative bleeding, operative time, duration of postoperative hospital stay, intraoperative complications, mortality, and morbidity.

**Results:**

Of the patients, 34 (% 56.6) were female and 26 (43.3%) were male, and the range of the age was between 33 and 78, and the mean age of the patients was 52.73 ± 11,617. While the amount of intraoperative bleeding was 321.864 ± 575.00 ml in the conventional group, this was found to be 370.31 ± 238.456 ml in the LigaSure group (p = 0.007). In the statistical evaluation of the operative time, the mean duration was determined as 310.00 ± 24.795 minutes in the conventional group, whereas it was determined as 265.16 ± 31.353 minutes in the LigaSure group (p = 0.001).

**Conclusions:**

The use of LVSS was associated with a significant reduction in the operative time and the rate of intra-operative complications.

## Background

Today, esophageal cancer can be best treated by the surgical methods. Worldwide esophageal cancer is one of the most deadly cancers. Surgical therapy remains as a main treatment method of esophageal cancer, and it is the best palliative procedure for the patients with dysphagia. The factors such as excessive intra-operative bleeding, blood transfusions, splenectomy procedures, and longer operative times lead to higher incidence of perioperative complications compared to extensive lymph node dissection [1]. In most research studies on esophageal cancer, the excess number of metastatic lymph nodes, higher rate of positive lymph nodes, amount of perioperative blood transfusion, tumor size, histological type, stage of tumor, and the need for respiratory support in the postoperative period are reported to be important prognostic factors [2].

Minimally invasive surgical procedures are associated with the use of advanced technological tools in operations. One of them is the LigaSure Vessel Sealing System (LVSS). This advanced system is being used instead of surgical sutures, clips, and the other methods of vascular ligation and providing hemostasis. The collagen and elastin in the vessel wall are denatured using LVSS, and a form of reinforced bipolar electrocautery that allows permanent fusion of the vessel wall by forming a hemostatic plug is used in vessels up to 7 mm in diameter [3,4]. Unnecessary bleedings can be prevented with the closure of the tissues with LigaSure without dissection or isolation of the vessels. The vessels that are closed with this system have been reported to be resistant to pressure up to three times the normal systolic pressure [5-7].

The efficiency of LVSS has been assessed in general surgery, urologic surgery, gynecologic surgery and thoracic surgery, and this system was found to reduce operative time, blood loss, damage of the adjacent tissues, and adhesions and facilitate the operation in open and closed operations [8,9].

In this study, we investigated the effect of LVSS, which has been routinely used for the patients with esophageal cancer in our clinic since 2006, on the operative time, intraoperative blood loss and intraoperative complications, and LVSS has been compared retrospectively with methods for hemostasis and tissue dissection techniques, which we have been used conventionally before 2006, in terms of differences and benefits. We were intended to evaluate whether the use of LVSS technique in esophagectomy would improve advantages compared with the conventional surgical procedure of esophageal cancer.

## Methods

Patients who underwent Ivor-Lewis esophagectomy for esophageal cancer between 2004 and 2011 were included in this study. 28 patients, who underwent surgery with clamp dissection and ligation between January 2004 and August 2006, and 32 patients who underwent Ivor Lewis esophagectomy using LigaSure between August 2006 and August 2011 were compared retrospectively. In our study, all patients underwent intra-thoracic esophagectomy and esophagogastrostomy. In all cases, intrathoracic anastomosis was achieved using a stapler. The patients who had manual anastomosis, who had anastomosis in the neck, who had transhiatal esophagectomy, and who had anastomosis via a left thoracoabdominal approach were not included in the study. The data form of each two groups were evaluated in terms of the amount of intraoperative blood loss, intraoperative complications, operative time, time to hospital discharge, postoperative complications, and mortality rates.

All patients were diagnosed histopathologically in the preoperative period. By using endoscopic biopsy, both histopathological evaluation and the determination of location and segment length of tumor were performed. The patients were also evaluated by radiological imaging methods. The patients who had radiologically detected metastases were assessed by the relevant departments for preoperative chemoradiotherapy. Only the patients who were operable in the preoperative period underwent surgery. All the lesions were located in the middle or distal part of the esophagus, in the esophagogastric junction, or cardia. All anastomoses were performed in the intrathoracic region. Taking care of the surgical margins and leaving a solid margin by at least 5 cm proximal to the tumor, anastomosis was performed at the level of thoracic inlet or at the level of azygos or at a lower level. A stapler was used for anastomosis.

After all the patients had had general anesthesia, they were intubated using a double-lumen endotracheal tube. Laparotomy was the first step of the operation. Abdomen was entered through a median incision above umbilicus. Stomach was mobilised using LigaSure or applying classical dissection by clamping and ligation. In both groups, a large amount of omentum was left around greater curvature and gastrectomy line was supported by the omental flap. Arterial arch (right gastroepiploic artery and vein) was preserved during mobilization of stomach and maximum effort was performed to avoid damage to the blood supply. In both groups, gastric sinistral artery was doubly-ligated and cut after the transfixion suture was placed. Abdominal lymph node dissection was performed, and the lymph nodes in the celiac region were removed. Hiatus was extended to provide a comfortable passage. Pyloric passage was controlled by finger, and pyloroplasty or pyloromyotomy, was applied in some cases, whereas no intervention was performed in some cases. Some cases who underwent gastric mobilization by the conventional method, developed bleeding and damage during the spleen mobilization. In these cases, splenectomy was performed if hemostasis was not provided. Especially, patients with splenic injury required blood transfusion. In patients in whom LigaSure was used, greater and lesser curvature, gastrosplenic ligament, phrenoesophageal and gastrophrenic ligaments were easily mobilized without any bleeding. Before closing the abdomen, a drain was placed on splenic bed.

All patients underwent right thoracotomy after the completion of laparotomy, and the thoracic esophagus line was mobilized by using the LigaSure or conventionally by blunt and sharp dissections. Azygos vein was doubly-ligated, and then was clipped and cut. Mediastinal nodal dissection was done for the most accurate disease staging. Stomach was used in all cases as replacement organ. Stomach was used in all cases as a replacement organ. Proximal gastrectomy was performed by pulling the stomach into the thorax and gastrectomy line was supported by omental flap. Esophagectomy was performed at least 5 cm proximal to the tumor and the safety of surgical margins was confirmed by the frozen section procedure. After esophagectomy, a purse suture was placed in the esophageal lumen. In all cases, esophagogastrostomy was performed using mechanical circular staplers. A nasogastric tube was placed and remained for at least 3-4 days after surgery. After checking the anastomotic line, anastomotic line located in the posterior mediastinum was supported with pleural flap.

All of the patients were received total parenteral nutrition for 7 days in the postoperative period. At postoperative 7th day, leakage control was performed by methylene blue test or by contrast-enhanced esophagograpy. The patients with hypoalbuminemia received albumin-replacement therapy.

### Statistical analysis

Descriptive statistics of continuous variables were expressed as mean, standard deviation (St), and as minimum and maximum value, while categorical variables were expressed as the number and percentage. Student's t test was used to compare the two groups in terms of continuous variables. The comparison of the categorical variables was performed using the proportion Z test, and Fisher's exact test. Statistical significance level was considered as 5%, and calculations were performed using SPSS (ver: 13) statistical software.

## Results

A total of 60 patients who underwent Ivor Lewis operation were analyzed. Of the patients, 34 (56.6%) were female and 26 (43.3%) were male, aged between 33 and 78, and the mean age was found to be 52.73 ± 11,617. There was a female predominance (34/26). A significant portion of the patients (51 cases, 85%) had squamous cell cancer. Nine patients had adenocarcinoma (15%). The tumor was located in the middle part of the esophagus in thirty patients (50%) and distal esophagus in 24 cases (40%). The tumor was located in the cardia and at the gastroesophageal junction in six cases (10%) (Table 1).

**Table 1:** Tumour localization

Tumour localization	LigaSure Group	Conventional Group	p
Mid-esophagus	17	13	0.604
Distal esophagus	12	9	0.065
Cardia and gastro esophageal junction	3	3	0.999

According to the TNM (Tumor, lymph node, metastasis) classification, 2 patients had grade 1 (3.3%), 15 cases had grade 2a (25%), 19 patients had grade 2b (31.6%), and 24 patients had grade 3 (40%) disease (Table 2). There was no significant difference in tumor staging between the two groups and they were found to have similar characteristics.

**Table 2:** Type of tumours and staging

	LigaSure Group	Conventional Group	p
**Tumour type**			
**Squamous cell**	27	24	0.884
**Adenocarcinoma**	5	4	0.884
**Stage of cancer**			
**Stage-I**	2	-	0.144
**Stage- IIA**	8	7	0.999
**Stage-IIB**	10	9	0.941
**Stage-III**	12	12	0.673

The two parameters, which we observed a statistically significant difference between the two groups, were the operative time and the amount of intraoperative bleeding (Table 3). While the amount of intraoperative bleeding was 321.864 ± 575.00 ml in the conventional group, this was found to be 370.31 ± 238.456 ml in the LVSS group (p = 0.007). In the statistical evaluation of the operative time, the mean duration was determined as 310.00 ± 24.795 minutes in the conventional group, whereas it was determined as 265.16 ± 31.353 minutes in the LigaSure group (p = 0.001).

**Table 3:** The comparative results between conventional and LigaSure groups

		N	Mean	**Std. Dev**.	**Min**.	**Max**.	p
**Age**	Liqasure Group	32	52,91	11,624	33	76	0,903
	Conventional Group	28	52,54	11,818	36	78	
	Total	60	52,73	11,617	33	78	
**Operation time**	Liqasure Group	32	265,16	31,353	200	340	0,001
	Conventional Group	28	310,00	24,795	250	360	
	Total	60	286,08	36,150	200	360	
**Peroperative bleeding**	Liqasure Group	32	370,31	238,456	180	1550	0,007
	Conventional Group	28	575,00	321,864	250	1600	
	Total	60	465,83	296,461	180	1600	
**Postoperative stay**	Liqasure Group	32	13,72	3,285	10	28	0,612
	Conventional Group	28	14,14	3,135	10	26	
	Total	60	13,92	3,196	10	28	

Two patients in the conventional group and 1 patient in the LigaSure group were found to have cardiac arrhythmia (Table 4). 3 cases in the conventional group suffered intraoperative iatrogenic splenic injury requiring splenectomy (p = 0.067).

**Table 4:** Peroperatuar and postoperatuar complications

	Liqasure Group	Conventional Group	p
**Peroperative complications:**			
**Cardiac arythmia**	1	2	0.485
**Spleenic injury**	0	3	0.067
**Postoperative complications:**			
**Contralateral hydrothorax**	3	2	0.753
**Anastomotic leakage**	1	2	0.485
**Wound infection**	3	4	0.558
**Pulmonary embolism**	1	1	0.924
**Cardiac complications**	0	2	0.142
**Gastric dilatation**	2	4	0.308
**Pneumothorax**	0	1	0.309
**Hospital mortality**	1	2	0.485

When the patients were evaluated in terms of postoperative complications, 11 cases in the LigaSure group and 18 cases in the conventional group developed postoperative complications. Common complications seen in both groups were wound infection, pleural effusion, and gastric dilatation. In LigaSure group, only one patient died due to anastomotic leakage developed at postoperative 6th day, whereas in the conventional group, one patient died due to anastomotic leakage at postoperative 6th day and one patient due to pulmonary embolism at postoperative 14th. The last patient had two additional comorbid conditions including diabetes and hypertension. One patient in the conventional group, who was detected to have anastomotic leakage at postoperative 3rd day, underwent rethoracotomy, and approximately 1.5 cm sized aperture which observed in the proximal gastrectomy line was primarily repaired and the patient was discharged.

When patients were compared for the duration of hospital stay in the postoperative period, the patients were discharged from hospital within 10 to 28 days after surgery (mean 13.72 ± 3,285 days) in LigaSure group, and within 10 to 26 days after surgery in the conventional group (mean 14.14 ± 3,135) (p = 0,612).

## Discussion

Esophageal cancer is one of the most lethal cancers, and it constitutes 7% of all cancers of the gastrointestinal tract [10,11]. It is the sixth most common cancer worldwide [12]. There has been marked improvement in the treatment and prognosis of esophageal cancer in recent years. Geographic distribution of esophageal cancer varies between different regions of the world. In our country, it is endemic in the Eastern Anatolia region, especially in Erzurum and Van. The incidence of esophageal cancer in our region and in the Eastern Anatolia region is similar to that of Iran. In our region gastric cancer is the most and second most common cancer in men and women respectively and esophageal cancer is the most common and fifth most common cancer in women and men respectively [13]. This cancer is 2-4 times more common in men than in women in Western societies; while it is more common in women in our region [14]. Our study also supported this female predominance. This situation may be associated with the preparation of many foods such as bread and meat at home, and by the fact that women are more likely to be exposed to biomass smoke.

Esophageal cancer consists of two major histopathological types: Squamous and adeno carcinomas. Adenoid cystic carcinoma, mucoepidermoid carcinoma, adenosquamos carcinoma, undifferentiated carcinoma, and malignant melanoma occur less frequently. These rare types are associated with very poor prognosis. While the incidence of adenocarcinoma has been increased especially in western societies over the past three decades, the incidence of squamous cell cancer remained unchanged [15-17]. However, we observed in our society and especially in our region that there has been no change in the incidence of squamous cell cancer, which is the most frequent one.

Esophageal cancer is a highly invasive cancer, which is associated with lymph node metastases in mediastinum, abdominal cavity, and neck [18]. Surgical resection is the best method in the palliative treatment of dysphagia, and offers the best chance of recovery, therefore it is still the best treatment for esophageal cancer. Adjuvant chemotherapy has been demonstrated to have no long term survival advantage in most randomized trials to date [19]. Therefore, surgical resection remains the only treatment method which occurs the best chance for recovery. However, the surgical treatment carries significant risks of morbidity and mortality. The mortality of 30-day rate after esophageal surgery reported in the literature was between 4%-10.7% [20-23]. Hospital mortality rates have been reported to range from 5.9 to 14% [20,21,23]. One of our patients who died has had splenectomy before. Advanced surgical techniques and improvements in anesthetic techniques and intensive care have positive impact on short-term survival and reduce the 30-day mortality and hospital mortality rates. Surgical techniques to be used in esophageal cancer surgery play an important role in the prevention of intraoperative and postoperative complications. Regardless of surgical procedure, the main purpose of surgical treatment is to minimalize perioperative complications, to reduce local recurrence rate, to increase 5-year survival rates and to increase quality of life in the postoperative period.

Today, esophageal cancer can be treated by a variety of surgical approaches. Ivor Lewis esophagectomy remains one of the most popular surgical methods today, because this surgical approach allows us to reach all of the above-mentioned lymph nodes, providing a full range of vision. With the use of state of the art technological products, this technique, which is the most preferred technique in our clinic, reduces intraoperative and postoperative complications and significantly increases therapeutic efficiency. The mortality rates reported with Ivor Lewis esophagectomy range from 1.4 to 6.8% and the complication rates range from 29 to 75% [24]. One of the major benefits of this procedure is that the bleeding is far less than other surgical procedures; therefore the negative effects of blood transfusions can be eliminated. So, we think, therefore, that this procedure is quite reliable. It provides direct visualization of mediastinum and excellent exposure, therefore, provides a better lymph node dissection and minimizes the bleeding. Ivor Lewis procedure was performed in all patients in our study in all cases, and the lymph node dissections of the two regions (abdomen and thorax) were performed within the field of direct vision.

Today, the use of minimally invasive methods is associated with more successful results in esophageal surgery. Postoperative mortality rates were reduced and 5-year survival rate after esophagectomy exceeded 25% with the higher rates of complete resection [25].

In both abdominal and thoracic stage of esophageal cancer surgery, manual ligation and cutting of vascular structures and organ dissections lead to both loss of time and unwanted bleedings. This situation leads to increased morbidity and mortality. One of three patients, who had mobilization of stomach with conventional method, and who had to be undergone splenectomy due to splenic damage and unwanted bleedings, has died. In some cases, it can be difficult to control the upper pole of the spleen especially during vasa brevia dissection. This also leads to undesirable damage of the spleen. In our cases we observed an increase in cardiovascular complications due to prolonged operative time. We have been using LVSS in our clinic since 2006. We use LVSS to mobilize the stomach except for gastric sinistral artery and vein. In the same way, we also safely use this method in the mediastinal dissection and in mobilising the thoracic esophagus, and in the tumor resection. LVSS is shown to be used safely in all vascular structures up to 7 mm in diameter. This technique is shown to reduce the operative time and the amount of intraoperative bleeding [26-28].

Over the past decade there have been many significant changes in the management of esophageal cancer. Several surgical procedures are available to resect esophageal carcinoma and restore the digestive system. The surgical procedures are based on concepts sometimes radically opposed with respect to the carcinologic approach: from transhiatal esophagectomies without node resection as described by Orringer et al. [29] to en block resections supported by Skinner [30]. To date Akiyama et al. [31] described the three-field colo-thoraco-abdominal lymph node dissection. Ivor Lewis procedure [32] combines an extended resection of the esophagus with either standart or extensive thoracic and abdominal lymph node dissection. Western and Eastern countries cause various attidues on their surgical management. In addition to the above surgical approaches recently reported thoracoscopic approach [33]. Minimally invasive esophagectomy by thoracoscopy, laparoscopy, and servicotomy has been described by several authors [34]. Recently, Cadiere et al. [34] has been described Ivor Lewis esophagectomy with manuel esogastric anastomosis by thoracoscopy in prone position and laparoscopy. With this technique, less pain, shorter hospital stay, rapid recovery was achieved. David EA et al. [35] modified a thoracoabdominal positioning for Ivor Lewis esophagectomy. In addition to this hybrid position, they also use a vertical mid-axillary thoracotomy fort he thoracic approach. This technique is useful for a number of reasons. This position allows for simultaneous manipulation within both cavities for delivery of the conduit. Campos et al. [36] have found that constructing a circular-stapled anastomosis with the trans-oral anvil allows for a standardised esophago-gastric anastomosis. This is a straightforward and reproducible technique tahs is particularly suited to the minimally invasive thoracoccopic approach, and has a low leak and stricture rate. There are new surgical techniques and surgical approach. Surgical technique is an important factor in preventing intraoperative and postoperative complications. For example recently, the use of self expandable metallic coated stents resulted in considerable improvements of thoracic anastomotic leaks [37]. Also, supporting the anastomosed region with omentum, pleura, pericardium, and fat tissue further decreases anastomotic leaks. The use of LVSS, reduced perioperative bleeding and prevent unplanned splenectomy.

In this study, the mean operative time for patients who underwent Ivor Lewis esophagectomy was 310.00 ± 24,795 minutes (250 to 360 minutes), whereas it was 265.16 ± 31,353 minutes (200 to 340 minutes) for patients who underwent LVSS (p = 0.001). While the amount of intraoperative bleeding was 321.864 ± 575.00 ml (250 to 1600 ml) in the conventional group, this was found to be 370.31 ± 238.456 ml (180 to 1550 ml) in the LigaSure group (p = 0.007). Therefore LVSS was associated with shorter operative time (average 55 minutes) and less bleeding (average 205 ml). Although it is not statistically significant, a relative reduction in the incidence of complications in patients underwent LVSS is also noteworthy.

## Conclusions

We think that the incidences of intraoperative and postoperative morbidity and mortality are lower and the survival rates are higher in patients who underwent esophagectomy with the LVSS and other advanced surgical techniques. The use of LVSS was found to provide significant reductions in operative time and the rate of intra-operative complications.

## Competing interests

The authors declare that they have no competing interests.

## Authors' contributions

FS, UC and AS acted in coseption and design. AS and UC acted in data analysis and interpretion. FS, and AS acted in manuscript writing. FS acted in revision of the article. All authors read and approved the final manuscript.
